# The Effectiveness of Topical Keratolytics (Alpha Hydroxy Acids/Beta Hydroxy Acids/Urea) in Treating Keratosis Pilaris: A Review of the Literature

**DOI:** 10.7759/cureus.100507

**Published:** 2025-12-31

**Authors:** Eleftheria Dampa

**Affiliations:** 1 Internal Medicine, Kilkis General Hospital, Kilkis, GRC

**Keywords:** follicular hyperkeratinization, glycolic acid, hyper-keratosis, keratin plugging, keratolytic treatment, keratosis pilaris, lactic acid, topical salicylic acid, urea, xerosis

## Abstract

Keratosis pilaris (KP) is a highly prevalent, benign disorder of follicular keratinization characterized by rough keratotic papules and variable perifollicular erythema, most commonly affecting the extensor upper arms, thighs, and buttocks. Although medically harmless, KP is frequently associated with cosmetic distress, reduced self-confidence, and persistent dissatisfaction with skin texture and appearance. The fundamental pathological process involves follicular hyperkeratosis with retention keratosis and abnormal desquamation, often occurring in the context of xerosis and epidermal barrier dysfunction, including associations with atopic dermatitis, ichthyosis vulgaris, and filaggrin-related barrier impairment. These mechanisms provide a strong rationale for topical keratolytic therapy, which aims to reduce corneocyte cohesion, facilitate desquamation, soften keratin plugs, and improve hydration of the stratum corneum. Topical keratolytics remain reasonable first-line, symptom-directed options for KP, with AHAs, BHAs, and urea all demonstrating potential benefit. However, the overall evidence base is constrained by small sample sizes, heterogeneous outcome measures, limited blinding, and short follow-up, with sparse long-term maintenance data and limited differentiation between texture-dominant and erythematous KP phenotypes.

## Introduction and background

Keratosis pilaris (KP) is a common benign disorder of keratinization characterized by perifollicular keratotic papules, typically distributed over the outer surfaces of the arms, thighs, and buttocks, and occasionally the face. KP frequently leads to patient concern due to its rough texture, erythema, and chronic, unresponsive treatment. The condition is highly prevalent and widespread, affecting an estimated 50-80% of adolescents and up to 40% of adults, with onset commonly occurring in early childhood and often diminishing with age [1].

The pathogenesis of KP is characterized by abnormal follicular keratinization leading to retention hyperkeratosis and blocking of the follicular ostia. Histological studies reveal orthokeratosis of the follicular ostium and infundibulum, dilated follicular infundibulum, follicular plug, dermal perivascular infiltration, and perifollicular scaling [2]. Genetic factors appear to play a significant role, with KP commonly linked to filaggrin (FLG) loss-of-function mutations and coexisting disorders of epidermal barrier dysfunction, such as atopic dermatitis and ichthyosis vulgaris [3]. Disruption of the skin barrier and altered desquamation contribute to the characteristic roughness and prominent keratotic papules [4].

Although harmless and benign in nature, KP can drastically affect quality of life. Patients commonly report cosmetic dissatisfaction, embarrassment, and persistent worry about the redness and texture of the skin. Due to the chronic nature of the symptoms and the lack of curative therapy, patients frequently seek topical treatment agents aimed at improving the appearance and feel of the skin. Topical keratolytics, including alpha-hydroxy acids (AHAs), beta-hydroxy acids (BHAs), and urea, are widely used as first-line agents, as they promote exfoliation, hydration, and normalization of keratinization. These agents have been employed to alleviate follicular plugging and smooth the stratum corneum, yet the evidence base supporting their relative effectiveness remains scattered across small trials and observational studies [5].

The purpose of this review is to synthesize and critically evaluate the current literature on the effectiveness of topical keratolytics, specifically AHAs, BHAs, and urea, in the management of KP. By summarizing available clinical evidence and identifying gaps in the literature, this review aims to guide clinicians in evidence-informed treatment selection and highlight areas requiring further investigation.

## Review

History and epidemiology of KP

KP is widely regarded as a common, benign follicular keratinization disorder that typically presents with small, rough keratotic follicular papules, most often on the extensor upper arms, thighs, and buttocks. Clinically, KP is often described as chronic and relapsing, with fluctuation over time and a tendency toward cosmetic concern rather than medical morbidity. Contemporary clinical references emphasize that KP most commonly affects children and adolescents, and many individuals experience partial improvement with age, though persistence into adulthood is also frequent [6]. One of the most comprehensive descriptions of the natural history of KP derives from the classic observational work by Poskitt and colleagues, which demonstrated that disease onset occurs predominantly in early life [7]. Specifically, 51% of cases began in the first decade, 35% in the second decade, 12% in the third decade, and 2% in the fourth decade of life. The same study identified common anatomical sites of involvement, particularly the upper arms, and reinforced the typical pattern of KP as a chronic condition with fluctuating severity over time rather than a transient dermatologic eruption. This natural history has important implications for clinical management and patient counseling, as the typically long-term course of the condition favors treatment strategies that are safe for repeated or intermittent use, such as topical keratolytic agents and emollients.

Pathophysiology of KP

KP mainly features follicular hyperkeratosis, a process in which excess keratin accumulates within the follicular infundibulum, leading to the formation of small, rough papules. Histopathological findings show expansion of the follicular orifice due to accumulation of keratinous material, occasionally containing coiled hair shafts [1]. This retention keratosis reflects an abnormal desquamation process where keratinocytes fail to shed properly, resulting in keratinous obstruction of the follicular opening.

Barrier dysfunction plays a central role in KP pathogenesis. Research studies suggest that patients with KP experience reduced skin hydration and increased transepidermal water loss (TEWL), consistent with an impaired epidermal barrier [8]. The condition has been associated with FLG loss-of-function mutations, classically implicated in skin barrier defects and atopic dermatitis, supporting the hypothesis that intrinsic barrier weakness predisposes to follicular plugging [3,9]. Barrier compromise also contributes to the characteristic xerosis seen in KP, which may exacerbate roughness, scaling, and follicular prominence.

KP frequently coexists with atopic tendencies, particularly atopic dermatitis and ichthyosis vulgaris [3]. This association is thought to stem from overlapping pathways involving impaired keratinocyte differentiation, reduced natural moisturizing factor, and suboptimal barrier repair [3,10]. Dry environments and low humidity further aggravate symptoms by intensifying epidermal dehydration and enhancing keratin retention [11].

The theoretical basis for using topical keratolytics (such as AHAs, BHAs, and urea) arises directly from these pathophysiological mechanisms. By promoting exfoliation and improving hydration of the stratum corneum, keratolytics counteract follicular hyperkeratosis and restore a smoother texture. Their ability to soften keratin plugs, enhance desquamation, and improve barrier function aligns closely with the underlying defects in KP, providing a mechanistic justification for their widespread clinical use.

Overview of keratolytic agents

Topical keratolytic agents constitute the cornerstone of first-line therapy for KP, targeting the fundamental abnormality of follicular hyperkeratosis. These agents act primarily by aiming at the reduction of corneocyte cohesion within the stratum corneum, thereby facilitating desquamation, smoothing follicular plugging, and improving skin texture. Keratolytic agents induce denaturation (via proteolytic cleavage) of keratin in corneocytes and corneodesmosomal junctions, eventually leading to different extents of structural and morphological thinning of the stratum corneum. Ultimately, the use of keratolytic agents leads to improvement in hyperkeratosis and a reduction in scaling [12]. Among the most commonly used keratolytic agents in KP are AHAs, BHAs, and urea, each of which exerts distinct yet complementary effects on epidermal differentiation and barrier function. Several of these substances and formulations have been used empirically for decades.

Alpha-Hydroxy Acids (AHAs)

AHAs, particularly glycolic acid and lactic acid, are water-soluble organic acids characterized by a single hydroxyl group attached to the α-carbon of the acid. These organic acids are commonly used in cosmetic formulations to remove thickened skin in hyperkeratotic disorders. Additionally, they are widely used in cosmetic products to address ultraviolet-related skin damage, having been shown to reduce skin roughness, solar keratosis, and excessive pigmentation, and to increase collagen levels and elastic fibre density [13,14]. Hydroxy acid-containing products are generally associated with improved exfoliation and increased moisturization [15]. Collectively, these processes promote regulated exfoliation and restoration of normal epidermal turnover, highlighting the relevance of AHAs in disorders involving aberrant follicular keratinization, such as KP. In the context of KP, AHAs are primarily employed to reduce follicular plugging and improve skin texture rather than to address underlying inflammation. *Glycolic Acid* Specifically, glycolic acid is capable of correcting abnormal follicular duct keratinization and removing excess keratinocyte buildup, in addition to promoting cutaneous metabolism and facilitating melanin metabolism. The use of glycolic acid has also been associated with improvement of scarring, positioning it as a viable therapeutic approach for KP [16]. Evidence suggests that the therapeutic efficacy of glycolic acid is concentration-dependent. In a five-year study, high-concentration glycolic acid was used to assess its efficacy and safety in the treatment of KP, with long-term follow-up extending to five years. The findings demonstrated significant improvements in skin roughness and follicular hyperpigmentation. Although the treatment was generally well tolerated, no significant difference was observed at the five-year follow-up compared with baseline [17]. The available literature largely characterizes AHA therapy for KP as part of combination treatment approaches, such as with retinoid cream [17]. A 2021 study demonstrated that high-concentration glycolic acid was effective in improving perifollicular erythema, papules, and pigmentation [17]. Enhanced efficacy was noted with higher concentrations and repeated treatments; however, these effects were not maintained over the long term [17]. *Lactic Acid* Lactic acid can be employed to manage papular lesions by promoting the degradation of dead skin cells, which helps to unclog pores and allows deeper penetration of adjunctive agents, including salicylic acid. Lactic acid primarily acts as a modulator of epidermal keratinization, while also functioning as a humectant, pH regulator, and mild irritant. Evidence suggests that lactic acid provides consistent therapeutic benefit in KP. In a 2015 study, patients with KP applied 10% lactic acid twice daily for three months, resulting in a 66% improvement in skin roughness, pigmentation, and overall appearance [8]. Increased skin conductance values were observed during treatment, and this improvement was maintained throughout the follow-up period. Treatment with 10% lactic acid resulted in early clinical improvement at four weeks, which persisted until the end of the 12-week treatment period [8]. Improvement in skin texture with 10% lactic acid cream is attributed to stimulation of epidermal cell migration toward the surface, potentially leading to slower development of stratum corneum hydration.

Beyond its exfoliative effect, lactic acid also plays a physiological role as a component of the natural moisturizing factor (NMF) within the stratum corneum. This dual keratolytic-humectant action is particularly relevant in KP, a condition frequently associated with xerosis and impaired barrier function. Increased stratum corneum hydration induced by lactic acid may decrease compensatory hyperkeratinization and facilitate softening of follicular plugs, thereby improving skin smoothness and patient satisfaction. Consistent application of lactic acid at concentrations ranging from 5% to 12% can reduce skin roughness and scaling, although reports indicate that even a 10% formulation may cause mild local irritation that is generally well tolerated [8]. *Beta-Hydroxy Acids (BHAs)*

In clinical dermatology, BHAs are almost entirely represented by salicylic acid. Similarly, in cosmetic formulations, the term “beta-hydroxy acid” specifically denotes salicylic acid. Unlike AHAs, salicylic acid is lipophilic, allowing it to penetrate lipid-rich environments such as the pilosebaceous unit, a key anatomical site involved in the pathogenesis of KP. Due to its lipophilic nature, salicylic acid facilitates the removal of intercellular lipids that are covalently associated with the cornified envelope of superficial epithelial cells [18]. Salicylic acid exerts its keratolytic effect primarily by solubilizing intercellular cement in the stratum corneum, thereby reducing corneocyte cohesion and promoting desquamation. This mechanism facilitates the clearance of compact keratin plugs obstructing follicular ostia, which are a histopathological hallmark of KP. Furthermore, due to its lipophilic properties and ability to traverse sebum, salicylic acid can dissolve debris, excess sebum, and follicular contents, allowing it to penetrate into hair follicles rather than acting exclusively on the surface epidermis. Through this mechanism, salicylic acid may target a key structural abnormality of KP more directly than exfoliants that act only at the epidermal surface. The antihyperplastic effect of salicylic acid on the epidermis has been widely employed by dermatologists for decades in chemical skin peeling, as well as in other dermatologic procedures. Salicylic acid is used across a wide concentration range, typically from 0.5% to 30%. Lower concentrations are primarily employed in the management of acne, whereas higher concentrations are mainly reserved for superficial facial chemical peeling [19,20]. Intermediate concentrations, generally between 3% and 6%, are commonly utilized in the treatment of hyperkeratotic disorders, including KP, psoriasis, and ichthyoses. Notably, Lookingbill and Marks have suggested a 6% salicylic acid formulation as a therapeutic option for KP [21]. In an open observational uncontrolled trial, KP demonstrated clinical improvement following once-daily evening application of a 6% salicylic acid multivesicular emulsion cream, used in conjunction with a designated moisturizer applied in the morning [22]. However, excessive concentrations or prolonged use of salicylic acid may impair epidermal barrier integrity by disrupting the skin’s natural protective function, removing essential lipids and viable corneocytes, and resulting in over-exfoliation. This may manifest clinically as irritation, erythema, or scaling and can paradoxically exacerbate the cosmetic appearance of KP if not appropriately balanced with adequate moisturization. Barrier disruption may further lead to the formation of microscopic fissures, increasing susceptibility to TEWL and penetration of external irritants.

Despite its widespread use in clinical practice, direct high-quality evidence evaluating salicylic acid monotherapy in KP is limited. Most published data consist of small clinical studies, expert reviews, and extrapolation from broader keratolytic literature. Nonetheless, salicylic acid remains a commonly recommended option in dermatology texts and reviews for KP due to its established keratolytic efficacy, follicular penetration, and familiarity among clinicians. Its role is best conceptualized as symptomatic and cosmetic, improving skin texture rather than altering the natural course of the condition. *Urea*

Urea is a naturally occurring component of the skin’s NMF and plays a central role in maintaining epidermal hydration and barrier homeostasis, supporting both adequate water retention and structural integrity of the stratum corneum. Urea is extensively utilized in dermatology for its ability to enhance skin barrier function and is among the most commonly used moisturizing and keratolytic agents. It enhances skin barrier function, including antimicrobial defense, by modulating keratinocyte gene expression involved in cellular differentiation and antimicrobial peptide synthesis [23]. In addition, it plays a critical role in the regulation of keratinocyte proliferation [24]. In dermatologic therapy, topical urea has long been used for a variety of hyperkeratotic and xerotic conditions, including ichthyosis vulgaris, xerosis, psoriasis, and KP. The effects of urea on the skin are concentration-dependent. At lower concentrations (≤10%), urea functions primarily as a humectant, enhancing water binding within the stratum corneum by increasing hygroscopicity and improving corneocyte flexibility. At higher concentrations (>10%), it exhibits keratolytic properties, disrupting hydrogen bonds within keratin filaments and softening compacted corneocytes [24]. Swanbeck provided the initial evidence supporting the keratolytic properties of urea in the 1960s [25,26], demonstrating that high-concentration urea formulations were effective in the treatment of ichthyosis and other hyperkeratotic disorders. This dual mechanism makes urea particularly well-suited for disorders characterized by abnormal keratin retention, such as KP.

Topical formulations containing 10-30% urea are commonly used in clinical practice. Preparations containing 10% are typically recommended for mild xerosis and maintenance therapy, whereas 20-30% formulations are reserved for more pronounced hyperkeratosis. Lookingbill and Marks have suggested a 20% urea formulation as a therapeutic option for KP [21].

Beyond its keratolytic and moisturizing actions, urea also contributes to barrier repair. Experimental and clinical studies have shown that urea can upregulate genes involved in epidermal differentiation, including FLG, loricrin, and involucrin, thereby supporting stratum corneum maturation and function [23]. This effect is particularly relevant in KP, which is frequently associated with filaggrin deficiency and impaired barrier integrity, especially in patients with coexisting atopic dermatitis or ichthyosis vulgaris. Clinical experience and observational data indicate that urea-containing formulations are generally well tolerated and may be suitable for long-term maintenance therapy in KP. A 2023 study reported that following four weeks of once-daily application of urea to the skin, most participants expressed satisfaction with skin texture, along with improved self-confidence and reduced skin-related embarrassment, with no significant adverse events observed [27]. Collectively, these findings support the tolerability and clinical suitability of a 20% urea cream for the management of KP.

Comparison between AHAs, BHAs, and urea

Overall Efficacy and Speed of Response

Across the limited interventional literature in KP, topical lactic acid (AHA) and salicylic acid (BHA) have the most direct comparative clinical data. In a randomized clinical study (split-side design) comparing 10% lactic acid to 5% salicylic acid applied twice daily for 12 weeks, both agents produced significant lesion reductions, with greater mean lesion reduction for lactic acid (66%) than salicylic acid (52%) by the end of treatment. Improvement occurred most rapidly in the first four weeks, then continued more gradually through week 12 [8].

Regarding the onset of clinically noticeable improvement, the most rapid response reported in published KP trials has been observed with 20% urea. In a four-week open-label clinical study, significant improvements in skin texture and smoothness were evident as early as one week and were sustained at four weeks, with the treatment demonstrating good tolerability [27]. High-strength glycolic acid (AHA) has also been studied in an open trial using 50-70% glycolic acid applied in repeated sessions (days 0, 20, 40, 60, 80), showing progressive improvement in papules and objective measures related to erythema/pigmentation over the treatment period, but no durable long-term benefit in the subset assessed at five years [17].

Tolerability and Irritation Risk

Tolerability is a major determinant of adherence because KP often requires ongoing, maintenance use. In a clinical study evaluating the safety and tolerability of lactic acid in comparison with salicylic acid, reported adverse effects were confined to mild, localized skin irritation [8]. Participants receiving lactic acid reported malodor and application-site discomfort, including burning or itching sensations, in the absence of visible systemic reactions. Although these symptoms occurred more often in the lactic acid group than in the salicylic acid group, the difference in irritation frequency did not reach statistical significance. Overall, adverse effects were limited to mild, localized irritation, with no systemic side effects observed.

In the case of urea, a study evaluating a 20% urea cream reported no significant adverse events, alongside high participant satisfaction and increased self-confidence after four weeks of use [27]. These findings are consistent with the broader dermatological literature, which indicates that urea functions primarily as a humectant at lower concentrations and exhibits progressively keratolytic properties at higher concentrations, with the risk of irritation increasing in a concentration-dependent manner [24].

The U.S. Food and Drug Administration (FDA) has advised caution regarding the use of products containing AHAs, as they may be associated with adverse reactions, including erythema, edema, burning sensations, and pruritus [28]. When applied at high concentrations, AHAs function as chemical peeling agents that disrupt corneocyte cohesion within the stratum corneum, leading to impairment of the skin barrier and subsequent irritation. In contrast, low concentrations of AHAs may exert beneficial effects on the skin, potentially mediated through epigenetic modulation of the inflammasome complex. Thus, AHAs demonstrate concentration-dependent, dual effects on cutaneous physiology.

Roughness and Erythema

Symptom clusters in KP vary among patients, with some primarily reporting a rough, “sandpaper-like” skin texture, while others are more troubled by perifollicular erythema. With respect to roughness and texture, all three agents (AHAs, BHAs, and urea) are plausible therapeutic options and are supported by varying degrees of clinical evidence. In a comparative study, both lactic acid and salicylic acid were associated with substantial reductions in lesion counts [8]. Additionally, a short-term clinical study demonstrated that 20% urea improved skin smoothness and texture, while an interventional study employing a clinic-style regimen showed that high-concentration glycolic acid led to improvements in skin roughness [8,17]. With regard to erythema, the available evidence is more limited and often derived from secondary outcomes. A study evaluating high-concentration glycolic acid explicitly assessed perifollicular erythema using hemoglobin-related measurements and demonstrated improvement over the course of treatment [17].

Overall comparison of AHA, BHA, and urea for KP

Key comparative considerations between AHAs, BHAs, and urea relevant to KP are summarized in Table 1, with emphasis on speed of improvement, tolerability, and the relative strength of available clinical evidence (Table 1).

**Table 1:** Comparison of AHA, BHA, and urea for keratosis pilaris (KP) AHA: Alpha-hydroxy acid; BHA: Beta-hydroxy acid.

Feature	AHA (e.g., lactic acid, glycolic acid)	BHA (salicylic acid)	Urea
Main mechanism relevant to KP	Reduces corneocyte cohesion → promotes desquamation/exfoliation	Lipophilic keratolytic; reduces corneocyte cohesion; follicular penetration is a common rationale	Humectant + barrier-support at lower %; keratolytic effect increases at higher %
Typical leave-on concentrations used in KP literature/clinical practice	Lactic acid 10% studied [8]; glycolic acid used from cosmetic strengths up to high-strength procedural applications	5% salicylic acid studied in KP trial [8]; common OTC leave-on 0.5–2% (varies by region)	10–20% common for roughness; 20% studied in KP [8]; higher % can be more keratolytic/irritant
Evidence in KP (direct clinical studies)	RCT split-side: 10% lactic acid improved lesions (66% mean reduction at 12 wks) [8]	RCT split-side: 5% salicylic acid improved lesions (52% mean reduction at 12 wks) [8]	Open-label clinical study: 20% urea improved texture at 1 and 4 wks; well tolerated [27]
Speed of Improvement (signal from studies)	Improvements seen by 4 weeks and continue through 12 weeks [8]	Improvements seen by 4 weeks and continue through 12 weeks [8]	Significant improvement reported at 1 week (noncomparative study), sustained at 4 weeks [27]
Tolerability (signal from studies)	Mild irritation; lactic acid reported more application discomfort/odor (burning/itching) [8]	Mild irritation; generally comparable or slightly better tolerated than lactic acid in trial [8]	Generally well tolerated in KP study; higher concentrations can sting/irritate in some [24,27]
Roughness vs Erythema	Stronger for roughness/texture; high-strength glycolic acid showed improvement in erythema-related measures in an interventional study [17]	Strong for roughness/follicular plugging [8]; erythema data limited	Strong for roughness + dryness; erythema improvements suggested in some regimen evaluations but less robustly proven [8]
Best “fit” Clinically	Texture-dominant KP; patients willing to tolerate some sting	Follicular plugging/roughness; acne-prone or oily areas may prefer	Dry, rough skin; maintenance-friendly regimens

Limitations and future directions

Although keratolytic agents are widely recommended for the management of KP, the current evidence base has several notable limitations. Randomized controlled trials (RCTs) are scarce, and existing studies are often small in scale, of short duration, and in some cases open-label. Outcome measures are heterogeneous, ranging from lesion counts and subjective assessments of skin smoothness to device-based measurements, thereby limiting the feasibility of meta-analyses and direct comparative evaluations. Long-term data supporting maintenance therapy are also limited; clinically, relapse or diminishing efficacy is common, and at least one interventional study of glycolic acid reported no significant difference at five-year follow-up in the assessed subset [17]. Furthermore, there is a paucity of evidence distinguishing treatment outcomes between texture-dominant and erythematous phenotypes of KP, despite their differing clinical behavior in practice. Despite KP being highly prevalent, the evidence base for topical keratolytics remains comparatively thin and heterogeneous. Existing clinical studies support improvement in texture/roughness with acids (e.g., lactic acid, salicylic acid, glycolic acid) and with urea-based regimens, but designs often involve small sample sizes, short follow-up, limited blinding, and variable outcome measures, making cross-study comparisons difficult. A clear priority is well-powered RCTs that compare (1) keratolytic vs vehicle, and (2) head-to-head comparisons between AHA vs BHA vs urea at commonly used concentrations. While a controlled clinical study has evaluated 10% lactic acid and 5% salicylic acid with instrumental assessment, broader replication across different KP phenotypes, body sites, and skin types is needed, along with standardized adverse-event reporting to better characterize tolerability [8]. In real-world practice, patients often use multi-ingredient regimens (e.g., keratolytic + moisturizer/barrier lipids; alternating acids; layered use with occlusion). Trials that test combination strategies, for example, urea (barrier/hydration + keratolysis) paired with low to moderate strength AHA/BHA could clarify whether combined approaches produce faster or more sustained improvements than monotherapy, and whether combinations increase irritation risk.

## Conclusions

KP is a highly prevalent disorder of follicular keratinization that, while benign, carries a meaningful cosmetic and psychosocial burden for many patients. Topical keratolytic agents remain the cornerstone of management, targeting the fundamental pathophysiological processes of follicular hyperkeratosis, impaired desquamation, and barrier dysfunction. Available evidence supports the clinical utility of AHAs, BHAs, and urea in improving skin texture and roughness, with lactic acid and urea demonstrating particularly consistent benefits and acceptable tolerability. Salicylic acid offers theoretical advantages related to follicular penetration but is supported by more limited direct evidence in KP. Across agents, therapeutic effects are primarily cosmetic and require ongoing use, as no intervention alters the natural course of the condition. Despite widespread clinical use, the current literature is constrained by small study sizes, heterogeneous outcome measures, and a lack of long-term comparative data. Future well-designed RCTs, standardized severity scoring systems, and studies evaluating combination regimens are needed to better define optimal treatment strategies. Until such data are available, individualized therapy based on symptom profile, tolerability, and patient preference remains the most pragmatic approach to managing KP.
